# Outbreak of Influenza and SARS-CoV-2 at the Armed Forces of the Philippines Health Service Education and Training Center, September 25–October 10, 2023

**Published:** 2024-05-20

**Authors:** John Mark Velasco, Maria Theresa Valderama, Paula Corazon Diones, Susie Leonardia, Simon Alcantara, Khajohn Joonlasak, Piyawan Chinnawirotpisan, Wudtichai Manasatienkij, Chonticha Klungthong, Errol Roy Arellano, Carrol Mae Osia, Joy Magistrado-Payot, Paul Fajardo, Fatima Claire Navarro, Kathryn McGuckin Wuertz, Aaron Farmer

**Affiliations:** 1 Walter Reed Army Institute of Research–Armed Forces Research Institute of Medical Sciences, Bangkok, Thailand; 2 V. Luna Medical Center, Armed Forces of the Philippines Health Service Command, Quezon City; 3 Public Health Service Center, Armed Forces of the Philippines Health Service Command, Quezon City; 4 Office of the Surgeon General, Camp General Emilio Aguinaldo, Quezon City, Philippines; 5 University of the Philippines Manila, Ermita

## Abstract

In the last week of September 2023, a surge of influenza-like illness was observed among students of the Armed Forces of the Philippines (AFP) Health Service Education and Training Center, where 48 (27 males and 21 females; age in years: mean 33, range 27-41) of 247 military students at the Center presented with respiratory symptoms. Between September 25 and October 10, 2023, all 48 symptomatic students were evaluated with real-time reverse transcription polymerase chain reaction and sequencing for both influenza and SARS-CoV-2. Thirteen (27%) students were found positive for influenza A/H3 only, 6 (13%) for SARS-CoV-2 only, and 4 (8%) were co-infected with influenza A/H3 and SARS-CoV-2. Seventeen influenza A/H3N2 viruses belonged to the same clade, 3C.2a1b.2a.2a.3a, and 4 SARS-CoV-2 sequences belonged to the JE1.1 lineage, indicating a common source outbreak for both. The influenza A/H3N2 circulating virus belonged to a different clade than the vaccine strain for 2023 (3C.2a1b.2a.2a). Only 4 students had received the influenza vaccine for 2023. In response, the AFP Surgeon General issued a memorandum to all military health institutions on October 19, 2023 that mandated influenza vaccination as a prerequisite for enrollment of students at all education and training centers, along with implementation of non-pharmaceutical interventions and early notification and testing of students exhibiting influenza-like-illness.

**What are the new findings?:**

This report demonstrates a common source outbreak of influenza and SARS-CoV-2 among students of the AFP Health Service Education and Training Center. Potential contributing factors to the outbreak included low influenza vaccine coverage, mismatch with the clade of the influenza vaccine strain for 2023, close living conditions, in addition to other factors conducive to the transmission of respiratory infections.

**What is the impact on readiness and force health protection?:**

Conditions during military schooling, such as close living quarters and sustained personal interactions, can significantly increase risk of morbidity related to outbreaks of respiratory pathogens. Prevention measures including requiring vaccination prior to enrollment may mitigate outbreaks of respiratory pathogens.

## 1 BACKGROUND

Because influenza and SARS-CoV-2 share similarities in their modes of transmission as well as common symptoms and clinical presentation, they can be challenging to distinguish.^1^ Laboratory testing can help differentiate influenza from SARS-CoV-2 infection and inform clinical management. Accurate diagnosis is particularly important for patients admitted to emergency medical departments with suspected influenza, as well as determining the cause of a respiratory illness outbreak. Reports of co-infection of SARS-CoV-2 and other respiratory viruses, as well as bacterial and fungal infections, have been reported.^2,3^

Both viruses exhibit a propensity for rapid spread within confined settings, such as households and military barracks.^4^4 Conditions unique to military populations such as habitation in close quarters and sustained interactions during deployments can place those individuals at higher risk for respiratory disease outbreaks compared to the general population.^5,6,7^

The Walter Reed Army Institute of Research-Armed Forces Research Institute of Medical Sciences (WRAIR-AFRIMS) and the Armed Forces of the Philippines (AFP) began collaborations on influenza-like-illness (ILI) surveillance in 2008. This collaboration resulted in the establishment of the Philippines-AFRIMS Virology Research Unit (PAVRU) in Manila, one of the WRAIR-AFRIMS network of sentinel sites in Southeast Asia, on the grounds of the Victoriano Luna Medical Center Hospital (VLMC), a tertiary hospital of the AFP. PAVRU was instrumental in detecting emerging and re-emerging diseases including the first cases of the pandemic influenza A(H1N1) 2009 (influenza A[H1N1]pdm09) in the AFP and providing laboratory confirmation and containment of influenza A(H1N1)pdm09 in several AFP camps.^8^ Establishment of the AFP-AFRIMS Collaborative Molecular Laboratory in March 2011 further increased laboratory testing capability and research activities for other diseases important to the military, such as arboviral, vector-borne and diarrheal diseases, wound and blood-borne infections, in addition to characterizing multi-drug resistant bacteria. Existing collaborative relationships ensured PAVRU and the AFPAFRIMS Collaborative Molecular Laboratory were strategically positioned to assist the AFP during the SARS-CoV-2 pandemic. The AFP-AFRIMS Collaborative Molecular Laboratory was one of the first laboratories accredited by the Philippines Department of Health for SARS-CoV-2 testing in the country.^9,10^

During the COVID-19 pandemic influenza circulation declined globally,^11,12^ to the extent to which an influenza B lineage was reported as becoming extinct.^13,14,15^ As COVID-19 cases decreased due to nonpharmaceutical interventions (e.g. mask wearing, social distancing, cleaning of frequently-touched surfaces, frequent hand-washing with soap or use of hand sanitizers, closure of places where people gather, etc.), vaccination, and validated treatment options, the World Health Organization (WHO) announced in May 2023 that COVID-19 was no longer a public health emergency of international concern.^16^ Movement restrictions, non-pharmaceutical interventions and low natural exposure to respiratory viruses during this 3-year period^12^ may have created an environment conducive to respiratory disease resurgence and outbreaks due to decreased probability of occurrence by recent natural influenza infections and limited generation of more durable and cross-reactive immune responses.^17,18^

The risk of respiratory disease resurgence was evidenced during the last week of September 2023, when local Philippine newspapers reported an increase of ILI in several schools, prompting suspension of in-person classes.^19^ Concurrently, a surge of ILI among students of the AFP Health Service Education and Training Center (AFPHSETC) triggered an outbreak investigation. This report describes the results of that investigation of the respiratory outbreak at the AFPHSETC detected by the AFP-AFRIMS Collaborative Molecular Laboratory.

## 2 METHODS

Forty-eight students enrolled at AFPHSETC who presented with ILI, defined as objective or subjective history of fever (>99.50F; within 3 and 5 days from onset of fever for outpatients and inpatients, respectively) and cough or sore throat were tested as part of this outbreak investigation. Nasal and/or throat swabs were collected by hospital and study staff at the swabbing and triage area beside the VLMC emergency room. A standard form recorded demographic and clinical data, including, but not limited to, patient sex, occupation, age, town or city residence, date of fever onset, travel and exposure history, medical and vaccination history, signs and symptoms, and recent laboratory tests. The AFPHSETC Commandant advised the symptomatic students to have themselves tested. The students belonged to 7 different class cohorts with varying term durations—September and October, July through September, July through November, and June through December—that were coincident during the outbreak period.

One respiratory swab was tested using Quickvue influenza A+B rapid test (Quidel, CA, US) and a second respiratory swab was stored in universal transport media (Remel, KS, US) from which viral ribonucleic acid (RNA) was extracted using a QIAamp viral RNA mini kit (QIAGEN, US). The AFPAFRIMS molecular laboratory performed real-time reverse transcription-polymerase chain reaction (RT-PCR) for influenza and SARS-CoV-2 using methods described previously.^20,21^ Next generation sequencing (NGS) was performed on an iSeq100 instrument using the iSeq100 reagent kit version 2 (Illumina, US).

Viral RNA extracted from SARS-CoV-2-positive samples (Ct≤28) was used as a template for amplicon sequencing with ARTIC SARS-CoV-2 version 5.3.2 primers. For SARS-CoV-2 genome sequences analysis, the Burrows-Wheeler Aligner MEM algorithm (BWA-MEM v.0.7.17) was used for reference mapping, with the Wuhan-Hu-1 genome sequence (GenBank accession NC_045512.2) as the reference. Consensus sequences were generated using iVAR version 1.3.1^22^ with specified criteria: mapping quality threshold >=30, base quality >=30, and a minimum depth of coverage of 10. Lineage and clade assignments were determined using Pangolin version 4.3.1^23^ and Nextclade version 2.14.1.

For influenza A genome sequencing, viral RNA was extracted from all influenza A PCR-positive samples, and the RNA was used as a template for amplicon sequencing using a primer set previously described by Zhou et al.^24^ including an additional primer, MBTuni-12G(5-ACGCGTGATCAGCGAAAGCAGG).^24^ DNA libraries were constructed and multiplexed using an Illumina DNA prep kit and pooled prior to sequencing. For influenza genome sequences analysis, the hemagglutinin (HA) consensus sequences were generated using the same tools and criteria mentioned, with appropriate reference sequences selected from the GenBank database. The HA gene sequences were used to identify the genotype and clade with Nextclade version 2.14.1. Percentage of nucleotide and amino acid similarity among influenza HA sequence results were then compared to the WHO vaccine-recommended H3N2 vaccine strains for 2023.

Maximum-likelihood trees were constructed using IQ-TREE version 2.03 with 1,000 bootstrap replicates and the GTR+F+I and TVM+F+G4 models for SARS-CoV-2 and influenza trees, respectively. The phylogenetic trees were visualized using FigTree version 1.4.4.

The AFP Health Service Command (AFPHSC) Research Ethics Committee and the WRAIR Institutional Review Board approved the protocol.

## 3 RESULTS

Forty-eight (27 males and 21 females; age in years: mean 33, range 27-41) military students who presented with ILI, out of 247 students in total, were enrolled in the investigation, with 13 (27%) and 6 (13%) positive for influenza A(H3) only and SARS-CoV-2 only by real-time PCR, respectively, while 4 (8%) were co-infected with influenza A/H3 and SARS-CoV-2. Symptoms in addition to fever, cough, or sore throat are listed, according to laboratory diagnosis, in the **Table**. Only 4 (8%) and 7 (15%) of the 48 students had received the influenza vaccines for 2023 and 2022, respectively. Among the 4 students co-infected with influenza A/H3 and SARS-CoV-2, half (n=2, 50%) had symptoms other than fever, particularly difficulty of breathing (**Table**).

Two (4%) students with an initial diagnosis of acute viral infection required hospital admission, but did not require intubation, with 1 positive for influenza A/H3 and the other negative for both influenza and SARS-CoV-2. All students had received at least 2 doses of a SARS-CoV-2 vaccine.

**Figure 1:**
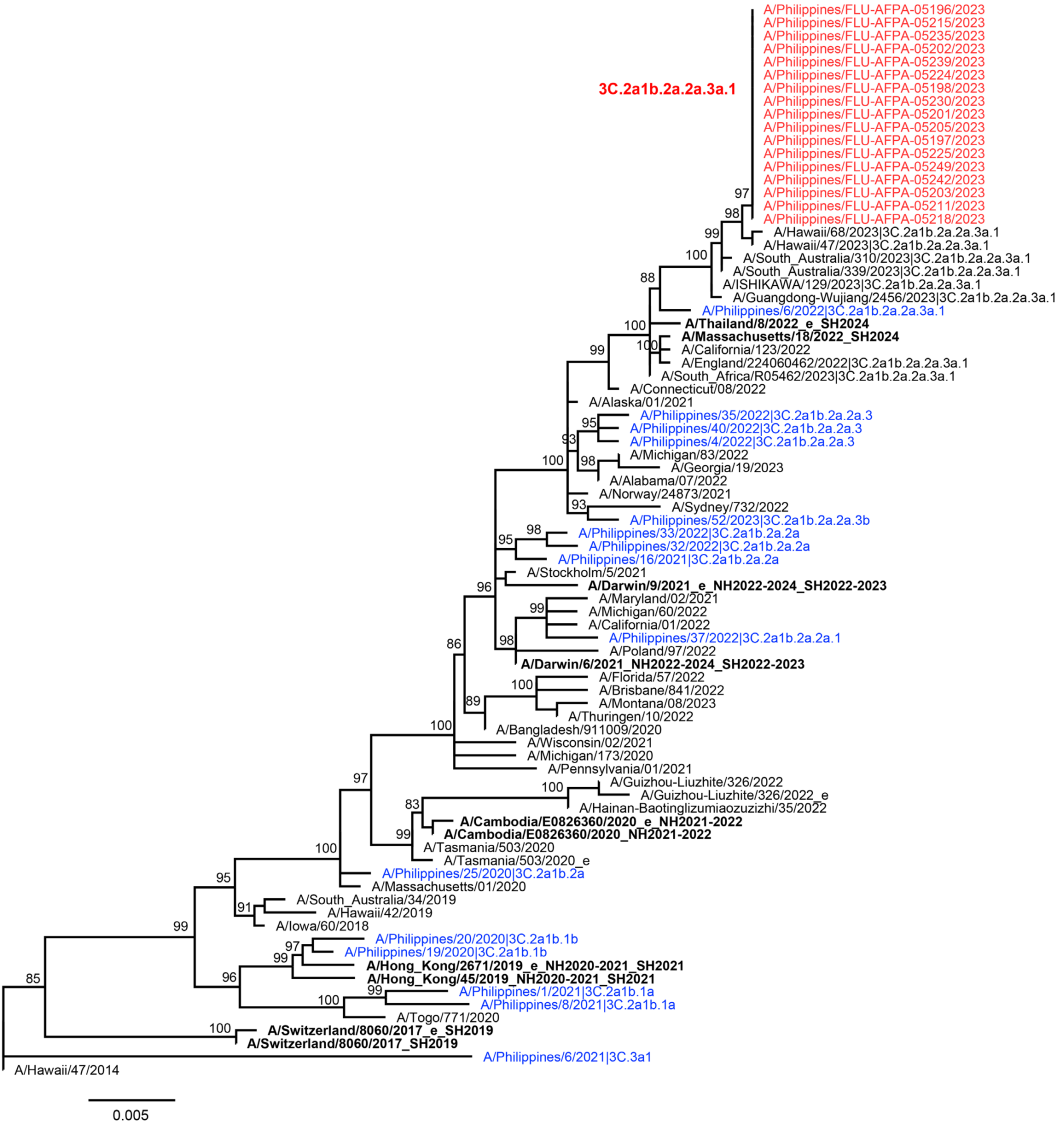
Maximum Likelihood Tree of 82 Influenza A / H3 HA Gene Sequences (1,701 nt) Including 17 New Sequences from the Philippines (red), and Sequences from GISAID and Genbank (15 sequences from the Philippines in blue, 10 sequences from WHO-recommended influenza A/H3 vaccine strains for 2019 to 2024 in bold black, and 40 sequences from other countries in black)

NGS of influenza A/H3 samples yielded the whole genome for 15 of 17 (88%) influenza RT-PCR-positive samples. Pathogen identification of influenza A/H3N2 and phylogenetic analysis using the HA gene of all 17 samples showed that they all belonged to the same clade, 3C.2a1b.2a.2a.3a.1 (**Figure1**). The clade of the influenza outbreak viruses differed from the clade of the WHO-recommended influenza A/H3N2 strains for the 2023 Northern and Southern influenza vaccines, A/Darwin/6/2021(H3N2)-like virus (cell culture or recombinant-based) and A/Darwin/9/2021(H3N2)-like virus (egg-based), respectively, which belonged to clade 3C.2a1b.2a.2a.

**Figure 2:**
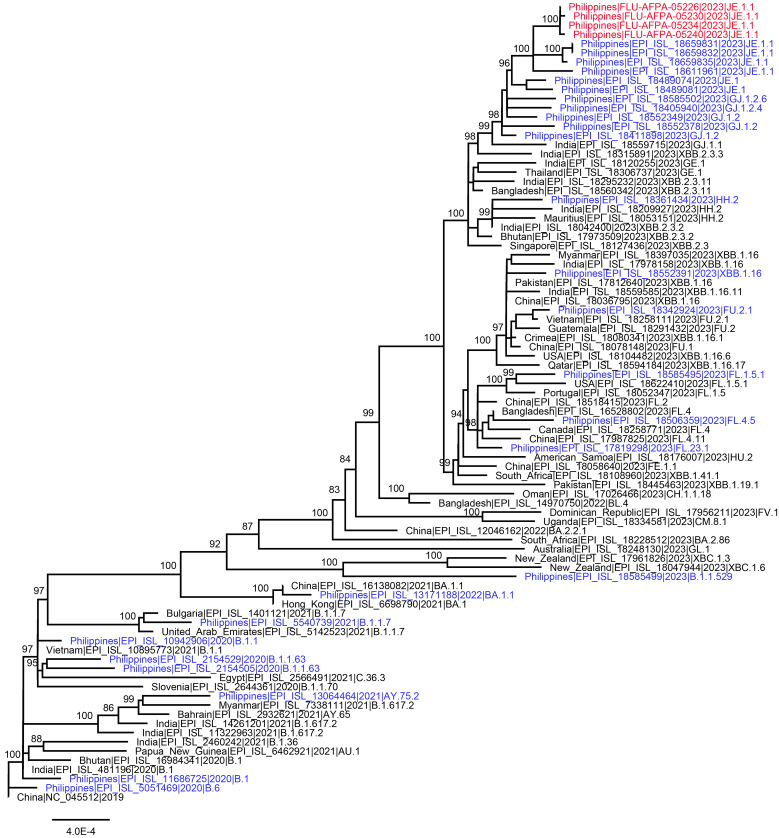
Maximum Likelihood Tree of 87 SARS-CoV-2 CDS Sequences (29,409 nt) Including 4 New Sequences from the Philippines (red), and 83 Sequences from GISAID and Genbank (27 sequences from the Philippines in blue and 56 sequences from other countries in black)

The percentage similarity of the influenza A/H3N2 outbreak viruses with the WHO-recommended influenza A/H3N2(A/Darwin/6/2021[H3N2]-like virus) strain for the cell culture or recombinant based Northern and Southern vaccine influenza vaccine for 2023 was 98.40% nucleotide and 97.79% amino acid similarity, respectively. The WHO-recommended influenza A/H3N2 Northern and Southern egg-based influenza vaccine strain (A/Darwin/9/2021[H3N2]-like virus) showed 98.03% nucleotide and 97.42% amino acid similarity, respectively. Sequencing of 4 of 10 SARS-CoV-2-positive samples showed that all belonged to the JE1.1 lineage (Pangolin) (**Figure2**) and 23E clade (clades.nextstrain.org).

Influenza and SARS-CoV-2 sampling and RT-PCR testing were completed on September 29, 2023 and October 2, 2023, respectively, and an initial report was sent to AFRIMS for review and confirmation of laboratory findings. The AFP Surgeon General was briefed on the investigation results on October 3, 2023, and on the following day (Oct. 4, 2023) a report was sent to the AFPHSETC Commandant, AFPHSC Commander, AFP Public Health Service Center (PHSC) Chief, VLMC Chief, and VLMC hospital infection control committee (HICC). PAVRU leadership briefed the Commandant of AFPHSETC, AFP PHSC Chief, and VLMC HICC Chief on October 9, 2023, after which measures such as mask wearing, social distancing, quarantining of symptomatic students were instituted.

Cases had begun to decrease in the first week of October 2023. NGS and bioinformatics analysis of influenza-positive samples were completed on October 10, 2023, allowing for review of any vaccine mismatch concerns. On October 19, 2023, the AFP Surgeon General issued a respiratory illness prevention memorandum addressed to the Chief Surgeon of the major services and the chiefs and commanders of major AFP health facilities. The memorandum included information on the respiratory outbreak and issued guidance for influenza vaccination as a prerequisite for enrollment of students at AFP education and training centers, implementation of preventive public health interventions (e.g., mask wearing, hand washing), early notification of ILI symptoms, and reporting of updated influenza and COVID-19 vaccination coverage.

## 4 DISCUSSION

Influenza and SARS-CoV-2 pose significant threats to public health and have far-reaching consequences for operational readiness and armed force strategic capabilities due to their rapid spread within units and high rates of morbidity. Distinguishing etiologic agents for respiratory illness is clinically difficult due to their similar signs and symptoms. The responsible pathogens of this outbreak were able to be determined rapidly by employing onsite AFP-AFRIMS Molecular Laboratory capabilities that enabled a wide variety of advanced molecular testing and NGS. By rapidly demonstrating that the outbreak was due to influenza A/H3N2 and SARS-CoV-2, additional targeted data (e.g., vaccination rates) could be obtained. The high influenza infection rates observed were most likely due to low influenza vaccination coverage.

This investigation was initiated as part of an ongoing study protocol that excludes testing of asymptomatic students, which could have underestimated actual infection rates. Co-infection with both influenza and SARS-CoV-2 was associated with increased morbidity, in particular difficulty of breathing (**Table**).

There was high nucleotide and amino acid percentage similarity of the influenza A/H3N2 outbreak viruses with the WHO-recommended influenza A/H3N2 strains for the cell culture or recombinant-based Northern and Southern vaccine influenza vaccine for 2023, but the clade of the influenza outbreak strains, 3C.2a1b.2a.2a.3a.1, did not match with the clade of the influenza A/H3 strains in the 2023 Northern and Southern hemisphere influenza vaccine strains (clade 3C.2a1b.2a.2a). This mismatch may have implications on vaccine effectiveness, especially if the mutations occurred in pivotal antigenic sites affecting glycosylation sites.^24^ Neither influenza A(H1N1)pdm09 nor influenza B were detected during this outbreak, but both subtypes and respiratory syncytial virus (RSV) have been observed in 2023 to be circulating, through our ongoing ILI surveillance (unpublished data). NGS results for influenza and SARS-CoV-2 indicated a combination of common source transmission, as all influenza A(H3)-positive samples and selected SARS-CoV-2 samples belonged to the same clade.

Timely and coordinated outbreak management is crucial for mitigating the impacts of both influenza and SARS-CoV-2. Minimizing the military implications from pathogens involves robust preventive measures, vaccination strategies, and effective surveillance to safeguard the health and operational capabilities of military forces. Rapid outbreak response and availability of confirmatory assays, which can identify the etiologic agent, are critical for both guiding immediate mitigation measures and formulating health policies to contain and prevent future outbreaks. Lessons from this report can inform strategies not only for future outbreak response but health policy formulation and targeted public health interventions, and can serve as a reminder of the importance of maintaining high vaccination rates with compatible vaccine strains.

Specimen collection involved nasal and throat swabs and not nasopharyngeal swabs, which may have affected assay yield and performance. The pathogen (or pathogens) causing respiratory symptoms among students who tested negative for both influenza and SARS-CoV-2 were not able to be determined. In addition, the clade/lineage of all SARS-CoV-2-positive samples were not able to be determined because some samples had low viral loads. Vaccine efficacy estimation was not performed due to low sample size and vaccination rates. Demographic, clinical, and vaccination data on the military students who did not present with ILI symptoms were unavailable, so comparisons to determine potential risk factors associated with infection could not be made.

This report underscores the need for increasing influenza vaccine coverage with well-matched vaccine strains, along with developing, maintaining, and sustaining rapid confirmatory testing capability, including pathogen discovery, for forward deployed laboratory sites. The 16-year, enduring collaboration and partnership of AFRIMS and the AFP made possible the rapid detection of this outbreak and subsequent translation of findings into actionable health policy. Rapid response capability is critical for timely detection and containment of outbreaks, as well as early detection of pathogens with potential to cause pandemics. Further testing with assays of broader detection capability for other respiratory pathogens is recommended.

## Figures and Tables

**Table 1:** Demographic, Clinical, and Vaccination Statuses of Students Included in the Outbreak Investigation^d^

	No.	%
Total	48	100
**Sex**		
Male	27	(56)
Female	21	(44)
**Age (average, range), y**	33 (27-41)	
**Influenza vaccination (year)**		
2023	4	(8)
2022	7	(15)
Unvaccinated	37	(77)
**Influenza A/H3 only**	13	(27)
Male	9	(69)
Headache, malaise/fatigue, runny nose, nasal congestion, generalized body pain/muscle ache, injected pharynx^a^	2	(15)^b^
Runny nose/nasal congestion^a^	2	(15)^b^
**SARS-CoV-2 only**	17 (16)	11 (10)
Male	1	(17)
Breathing difficultly, runny nose/nasal congestion^a^	1	(17)^b^
Runny nose/ nasal congestiona^a^	1	(17)^b^
**Co-infected with influenza A/H3 and SARS-CoV-2**	4	(8)
Male	2	(50)
Breathing difficulty, headache, malaise/fatigue^a^	1	(25)^b^
Breathing difficulty, headache, runny nose/nasal congestion, generalized body pain/muscle ache^a^	1	(25)^b^

Abbreviations: y, years; SARS-CoV-2, severe acute respiratory syndrome-associated coronavirus disease strain 2.

^a^Symptoms in addition to fever, cough, and/or sore throat.

^b^Percent among those who tested positive for the specified pathogen(s).

## AUTHOR AFFILIATIONS

Walter Reed Army Institute of Research–Armed Forces Research Institute of Medical Sciences, Bangkok, Thailand: Dr. Velasco, Ms. Valderama, Dr. Diones, Ms. Leonardia, Mr. Alcantara, Mr. Joonlasak, Dr. Chinnawirotpisan, Dr. Manasatienkij, Dr. Klungthong, Dr. McGuckin Wuertz, Dr. Farmer; V. Luna Medical Center, AFP Health Service Command, Quezon City: Dr. Arellano; Public Health Service Center, AFP Health Service Command, Quezon City: Dr. Osia, Dr. Fajardo; Office of the Surgeon General, Camp General Emilio Aguinaldo, Quezon City: Dr. Magistrado-Payot, Dr. Navarro; University of the Philippines Manila, Ermita: Dr. Velasco
